# “Patients with amyotrophic lateral sclerosis (ALS) are usually nice persons”—How physicians experienced in ALS see the personality characteristics of their patients

**DOI:** 10.1002/brb3.599

**Published:** 2016-11-10

**Authors:** Theresa Mehl, Berit Jordan, Stephan Zierz

**Affiliations:** ^1^ Department of NeurologyMartin‐Luther‐University Halle‐WittenbergHalle/SaaleGermany

**Keywords:** amyotrophic lateral sclerosis, five‐factor model of personality, personality

## Abstract

**Introduction:**

Physicians experienced in the treatment of patients with amyotrophic lateral sclerosis (ALS) occasionally describe these patients as “nice” persons.

**Methods:**

ALS experienced physicians (*n *=* *36) were asked to assess the personality characteristics of ALS patients using a multidimensional personality questionnaire based on the five‐factor model of personality. Control groups consisted of physicians experienced in Myasthenia gravis (MG) (*n *=* *21) and lung cancer (LC) (*n *=* *36).

**Results:**

In the dimension *Agreeableness* ALS patients were rated significantly higher than the other groups (*p *<* *.001). This was mainly due to the high scores for converse adjective pairs “stubborn—compliant” and “selfish—helpful”.

**Discussion:**

The dimension *Agreeableness* is very similar to “niceness”. Results support the anecdotal description of ALS patients as “nice” persons. Personality characteristics of ALS patients differentiate them from other patient groups. It remains open whether the “nice” personality structure is linked to the susceptibility to the disease.

## 1 Introduction

In studies and reviews on amyotrophic lateral sclerosis (ALS), physicians experienced in ALS sometimes give personal comments on the personality of these patients. They mostly describe these patients as “genuinely pleasant” (Wilbourn & Mitsumoto, 1998) and “pleasant and warm” (Borasio & Miller, 2001), who were unusually cheerful and stoic in view of their serious illness (Brown & Mueller, 1970). It also has been mentioned that “it is a privilege to work with ALS patients” (Borasio & Miller, 2001). It has even been suggested that the patients’ personality may serve as an unofficial exclusion criterion for medical and technical staff because the patient “is not nice enough” (Wilbourn & Mitsumoto, 1998). Despite these consistently positive remarks and informal observations from clinicians, there are only a small number of studies on specific personality characteristics of patients with ALS. In one study, the self‐assessment questionnaire “Minnesota Multiphasic Personality Inventory” was used to assessed the personality profiles of 38 ALS patients (Peters, Swenson, & Mulder, 1978). However, no specific character traits were found. In another study, not the ALS patients themselves but their caregivers (*n *=* *49), mostly their relatives, were asked to evaluate the patients premorbid personality using the third‐party version of the “NEO Personality Inventory” (NEO‐PI‐R) (Grossman, Levin, & Bradely, 2006). In this study ALS patients showed significantly higher values for the personality dimension called “Openness” compared to non‐ALS patients.

In the present study, a multidimensional personality questionnaire was used to analyze systematically how experienced physicians assessed the personality structure of ALS patients. In contrast to previous studies on patients themselves or their relatives (Grossman et al., 2006; Peters et al., 1978), we asked experienced physicians. The presence of an ALS‐specific personality could result in development of individual psychological treatment methods for dealing with these patients.

## 2 Patients and Methods

The personality characteristics of ALS patients were assessed by neurologists from neuromuscular centers in Germany using a shortened version of the “NEO Five‐Factor Inventory” (NEO‐FFI) (Borkenau & Ostendorf, 2003). This version consists of a total of 30 converse pairs of adjectives that represent the most basic dimensions of personality based on the established five‐factor model: *Neuroticism*,* Extraversion*,* Openness*,* Agreeableness*, and *Conscientiousness*. Consequently, there were six pairs of adjectives for each dimension. Clinicians were asked to rate for each pair using a five‐point score which best describes the “average” ALS patient (0 = strongly agree to the negative pole, 1 = agree to the negative pole, 2 = neutral, 3 = agree to the positive pole of the dimension, and 4 = strongly agree to the positive pole of the dimension). Thus, for every dimension a maximum score of 24 could be reached.

The control groups consisted of experienced physicians scoring the personality of patients with lung cancer (LC) and Myasthenia gravis (MG) in the same manner. LC patients were selected because of their similar poor prognosis as those of the ALS patients. Therefore, the possibility that the ALS‐experienced physicians assessed their patients more positive because of their dismal prognosis could almost entirely excluded. MG patients were selected because MG represents another neuromuscular but curable disease. The nationwide recruitment of the ALS and MG physicians was based on a list of neuromuscular centers published by the German Society for Patients with Muscle Disorders (Deutsche Gesellschaft für Muskelkranke). Because of possible bias, colleagues from our department were excluded from this study. The LC experts were recruited through the website of the “Association of Statutory Health Insurance Physicians” of Germany. This group consisted of specialists for medical oncology, pulmonology, and radiotherapy. All expert groups received the same personality questionnaire (shortened NEO‐FFI) by mail between October 2014 and August 2015. Additionally, a reminder letter was sent by mail after 4 weeks. In order to ensure an appropriate expertise with the particular patient group, the level of expertise was assessed as follows: (1) number of treated ALS patients per year (1 = less than 6 patients, 2 = 6–12 patients, 3 = more than 12 patients) and by (2) duration of practice (1 = less than 2 years, 2 = between 2 and 4 years, 3 = more than 4 years). Consequently, total score of experience could range between 2 and 6. The same scoring system was applied to the LC and MG experts.

### 2.1 Statistical analysis

A univariate analysis of variances (ANOVA) was calculated to investigate differences in physicians ratings of the five personality dimensions for ALS, MG, and LC patients. Normal distribution of data was tested using the Kolmogorov–Smirnov test. Possible deviations from the assumption of variance homogeneity were tested using the Levene test. Because of the multiple testing, a post hoc analysis with the conservative Scheffe's test was conducted with the relevant 95% confidence intervals (CI). This test performs a complex group comparison based on linear combinations and can also be used for groups of different sizes. *p*‐Values <.05 were considered significant. All tests were two tailed. Data were processed using the Statistical Package for Social Sciences program version 20. Missing data were not included in the statistical analysis.

### 2.2 Ethical standards

The manuscript does not contain clinical studies or patient data.

## 3 Results

A total of 60 questionnaires were sent by mail to each expert group, followed by a reminder letter. Altogether, 36 ALS experts (60%), 21 MG experts (35%), and 36 LC experts (60%) replied. The final ALS expert group consisted of 31 male (86%) and 5 female (14%) experts with a mean age of 49.17 years (range: 31–69 years). The MG expert group consisted of 15 male (71%) and 6 female (29%) experts with a mean age of 49.96 years (range: 32–65 years). The LC expert group consisted of 19 male (53%) and 17 female (47%) experts with a mean age of 44.25 years (range: 26–65 years). There were no significant age differences between the three groups. Gender distribution in the expert groups was not significantly different in ALS and MG expert group as well as in MG and LC expert group. Spearman rank correlation revealed no significant correlations between gender and the five tested personality dimension for each expert group. Level of clinicians expertise was high to very high (Scores 5–6) in 81% ALS experts, 100% of MG experts, and 78% of LC experts.

**Figure 1:**
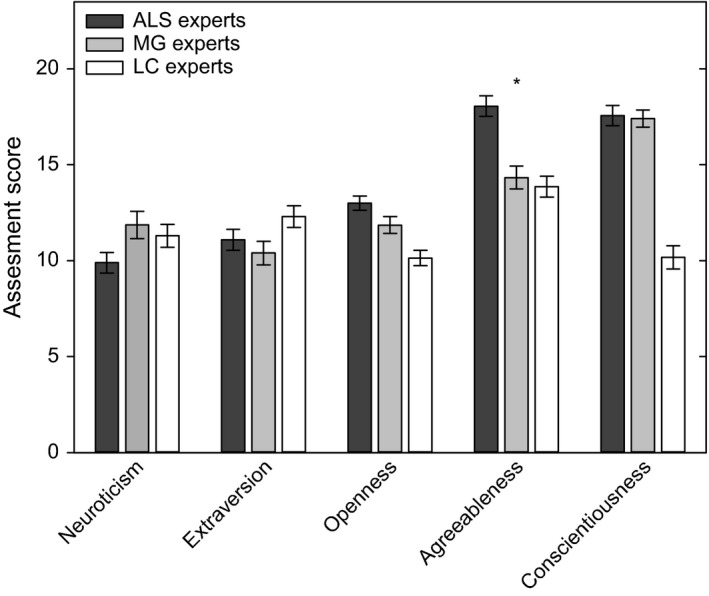
Physicians rating of the five personality dimensions of patients with amyotrophic lateral sclerosis (ALS;* n* = 36, black columns), with Myasthenia gravis (MG,* n* = 21, gray columns), and with lung cancer (LC,* n* = 36, open columns). The maximal possible score in each dimension is 24. Mean values ± standard errors are shown. Significantly higher scores for ALS compared to the other groups are indicated (*p < .001)

**Figure 2:**
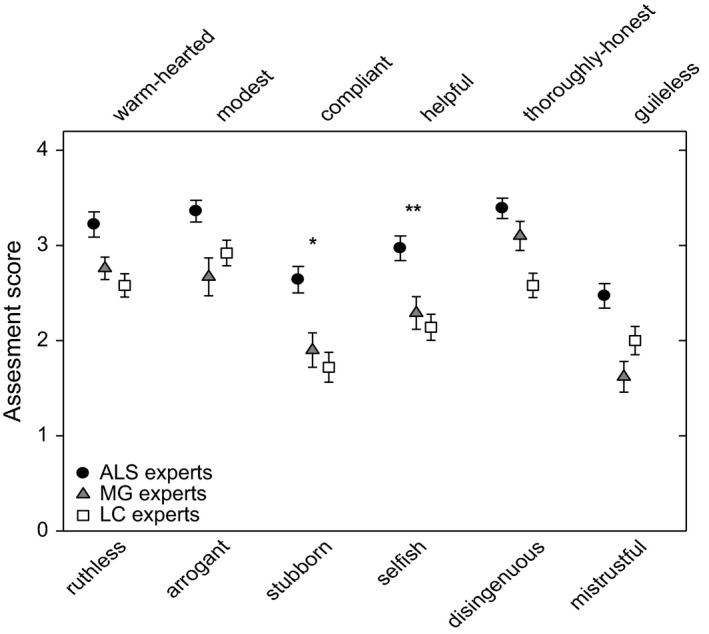
Physicians rating of the six items of the personality dimension *Agreeableness* of patients with amyotrophic lateral sclerosis (ALS;* n* = 36, black circles), with Myasthenia gravis (MG,* n* = 21, gray triangle), and with lung cancer (LC,* n* = 36, open squares). A score of zero indicates entirely agreement with the negative pole, and a score of 4 entirely agreement with the positive pole of each item. Mean values ± standard errors are shown. Significantly higher scores for ALS compared to the other groups are indicated (*p < .05, **p < .01)

Means and standard errors of the five personality dimensions are shown in Figure 1. Expert ratings were normally distributed and had homogeneity of variances. Univariate ANOVA revealed significant differences between the ratings of the patient groups for dimensions *Openness* (*F *=* *14.48, *p *<* *.001), *Agreeableness* (*F *=* *18.50, *p *<* *.001), and *Conscientiousness* (*F *=* *58.63, *p *<* *.001). The post hoc test showed that only the dimension *Agreeableness* was rated significantly higher for the ALS patients compared to the other patient groups (*p *<* *.001, CI 1.60–5.85). For the dimension *Conscientiousness*, the LC group was scored significant lower than the MG group (*p *<* *.001). As shown in Figure 2, the high score for the dimension *Agreeableness* (*M *=* *18.06, *SD* = 3.19) judged by ALS experts in comparison to LC (*M *=* *13.86, *SD* = 3.23) and MG (*M *=* *14.33, *SD* = 2.73) experts was in particular due to consideration of rated adjectives *compliant* versus *stubborn* (*p *=* *.012) and *helpful* versus *selfish* (*p *=* *.009). Both personality characteristics of compliance and helpfulness seem to predominate the positive perception of ALS patients behavior in comparison to MG and LC patients.

## 4 Discussion

The obtained results support the occasional statements that ALS patients are usually “nice” (Borasio & Miller, 2001; Wilbourn & Mitsumoto, 1998) persons. Niceness is not a dimension of the five‐factor model of personality, but it is very close to the dimension *Agreeableness*. Persons with high scores on the dimension *Agreeableness* can be described as warm, nice, and kindhearted persons who meet other persons with goodness and helpfulness (Ostendorf & Angleitner, 2004). Within the five personality dimensions, only *Agreeableness* showed an ALS‐specific significant difference. None of the other personality dimensions achieved statistical significance between the ALS and the other expert groups.

In a caregiver‐based (mainly relatives) study which evaluated the premorbid personality, ALS patients had a lower score on the dimension *Openness* compared to patients with LC (Grossman et al., 2006). A low score on that dimension describes a more factually oriented person with traditional and conventional values who tend to comply with the physicians instructions (Borkenau & Ostendorf, 2003). This increased willingness to follow the medical advice prompt clinicians to perceive the ALS patients as nicer and more cooperative than other patient groups. This assessment of caregivers is consistent with the assessment made by clinicians in this study.

However, it cannot be excluded that a feeling of compassion for the patients with the dramatic course of the disease influences the positive judgment of physicians and caregivers. In both studies, there was not such a positive assessment of the patients with LC that also have a poor prognosis. The study does not necessarily imply that similar results would be found in a direct survey of the patients themselves.

A possible limitation might be the use of a shortened version of the questionnaire NEO‐FFI. However, the applied questionnaire followed very close to the well‐validated NEO‐FFI and the adjectives to assess the five dimensions are defined in the official manual.

In conclusion, this study indicates that there seems to be a specific personality structure common in patients with ALS. It remains open if this personality is linked to the susceptibility of the disease.

## Conflict of Interest

The authors declare that they have no conflict of interest.
